# Ichoræmia

**Published:** 1892-03

**Authors:** C. R. Butler

**Affiliations:** Cleveland, O.


### Ichoræmia.

BY C. R. BUTLER, M.D., D.D.S., CLEVELAND, O.

Read before the Ohio State Dental Society, held at Columbus, December, 1891.

The subject of ichoræmia has excited of late the attention of the most careful investigators, so I venture a few thoughts on conditions of the mouth that affect the general system.

It is a common saying there is more or less trouble with the teeth from the cradle to the coffin.

Permit me to remark that dentition, while it is a physiological process, is one of continuous irritation. The family physician has opportunity to observe the effects of hyper dental irritation, and when it is in excess, the skill of the physician, surgeon and dentist is in demand to modify or control it, the same as in any other part of the body, and the case should be treated on general principles.

If it be of a local nature, showing a tense tumefaction of the gum over an erupting tooth, the lancet may be used with good effect, making a crucial cut if it be a molar, notwithstanding authors and practitioners widely differ.

It may be of a more general character and attributable to a faulty development of the osseous structure as a whole. If so tonics will be more efficacious than the lancet.

The weaklings are the ones that tax to the highest skill, and to this class we shall confine the most of our remarks. They come from the humble cottage and palatial home, with miserable structured teeth from first to last.

Of late considerable has been said about the application of the nitrate of silver in powder, of solid crystal to the partial enameled and decaying teeth, deciduous and first permanent molars. The practice is not all new, but the demand for something beyond the ordinary means warrants us in giving it more than an indifferent trial.

Many of these desperate cases only come to the notice of the physician and dentist when the suffering becomes unbearable from an exposed dying or dead pulp, ulcers in the mouth, and the formation of pus sockets about the teeth that become points for infection of no trivial character.

The sub-maxillary glands are often affected, and the internal carotid and internal maxillary arteries are ready channels for the distribution of septic poison, giving a train of symptoms familiar to us all.

Abscesses associated with the temporary teeth are a serious item, especially if the child be delicate or sickly. The germs of the developing permanent teeth may be destroyed, and necrosis of the maxilla in part result. Opening of the pus pocket or abscess, does not always close the trouble. Thorough washing out with phenol sodique, dilute, or any mild antiseptic, also drainage for a short time may be necessary.

Some might suggest immediate extraction would be demanded; that is not the best practice in many cases; the child needs teeth for mastication as well as the adult.

The etiology of the various lesions of the tooth structure is not definitely settled or demonstrated. And there is no provision yet discovered whereby a defect, disease, or fracture even, may be repaired, as in other bones, and yet they are vital organs.

In and about these diseased teeth septic matter is found, and extreme care should be exercised by the dentist or operator not to poison himself or others with the instruments that he is using daily.

From personal experience I may speak of the pain and prostration following a wound in the index finger of my left hand. In about three days after the inoculation there was great tenderness, with stinging pain in the finger extending up the arm into the back of the head. An abscess formed about the site of the wound that had to be opened. The dressing used was the corrosive sublimate, 1 to 1000; solution, in hot water. The general prostration continued for weeks.

Dr. W. D. Miller cites a case of chronic pyæmia as the resul of a wound by a dental instrument where hundreds of abscesses formed in different parts of the body

Cases have come under observation within a few months that showed symptoms of infection from unclean instruments in extraction.

Whether this infection be microbic or bacillic I am not here to assert or prove, but I am satisfied that poisoning does occur of a serious character.

"Trained nurses" is becoming quite the thing; they should have a clean healthy mouth and teeth, especially those that have the care of young children; also they should appreciate the importance of hygiene of the teeth and mouth if the child is afflicted with a wasting disease or protracted fever.

